# Supplementary figures and images for: Splicing deficiency is driven by genomic erosion in non-recombining algal mating-type chromosomes
Source: PLoS Biol. 2026 Jun 25;24(6):e3003823. doi: 10.1371/journal.pbio.3003823 (PMC13298755; doi:10.1371/journal.pbio.3003823)

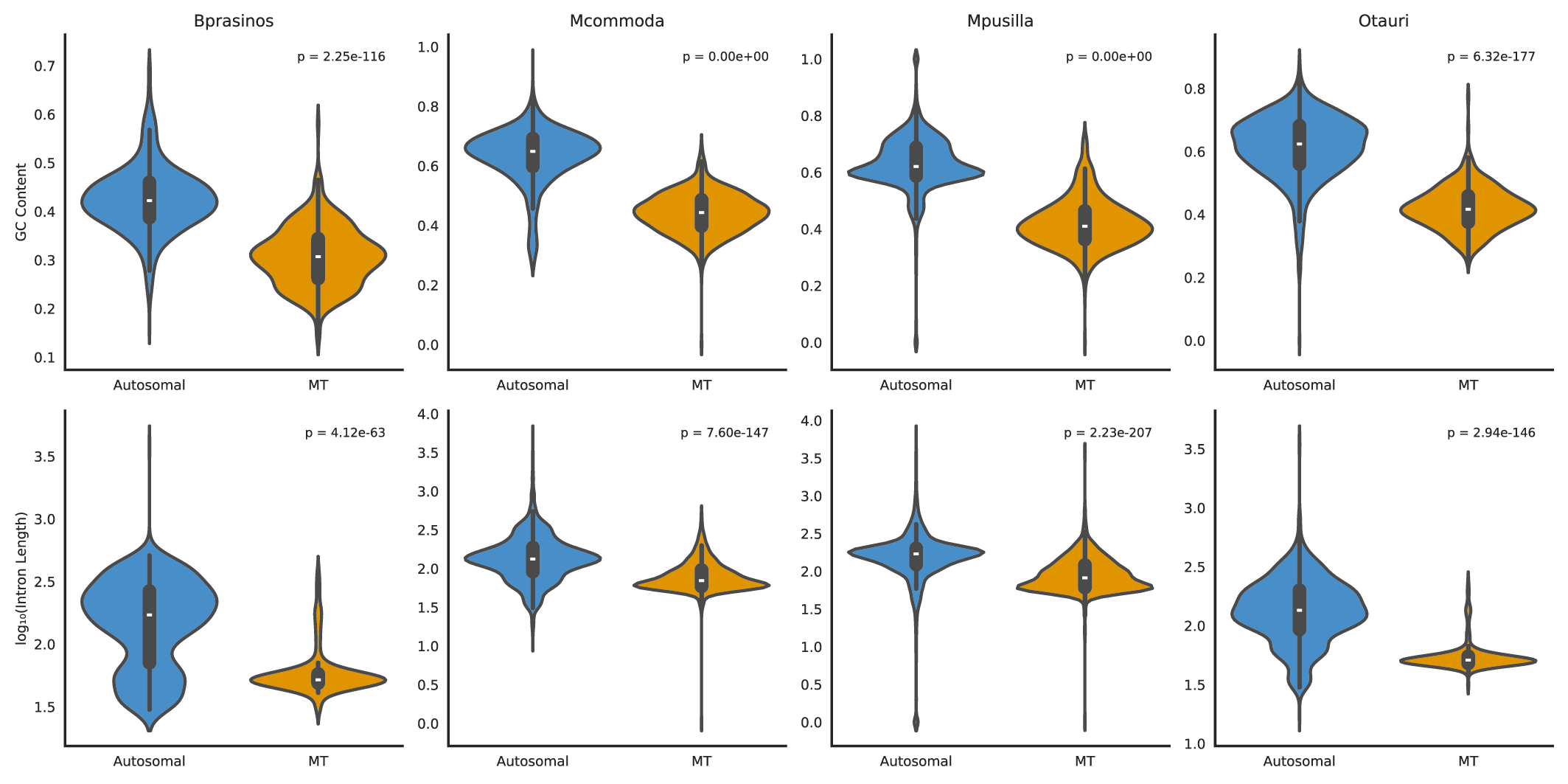

Supplement: S1 Fig — Underlying data for this figure are available in the GitHub repository at https://github.com/russcd/mating-type-missplicing (file: data/{species}.features.branchpoints.tsv). (TIF) [file pbio.3003823.s001.tif]

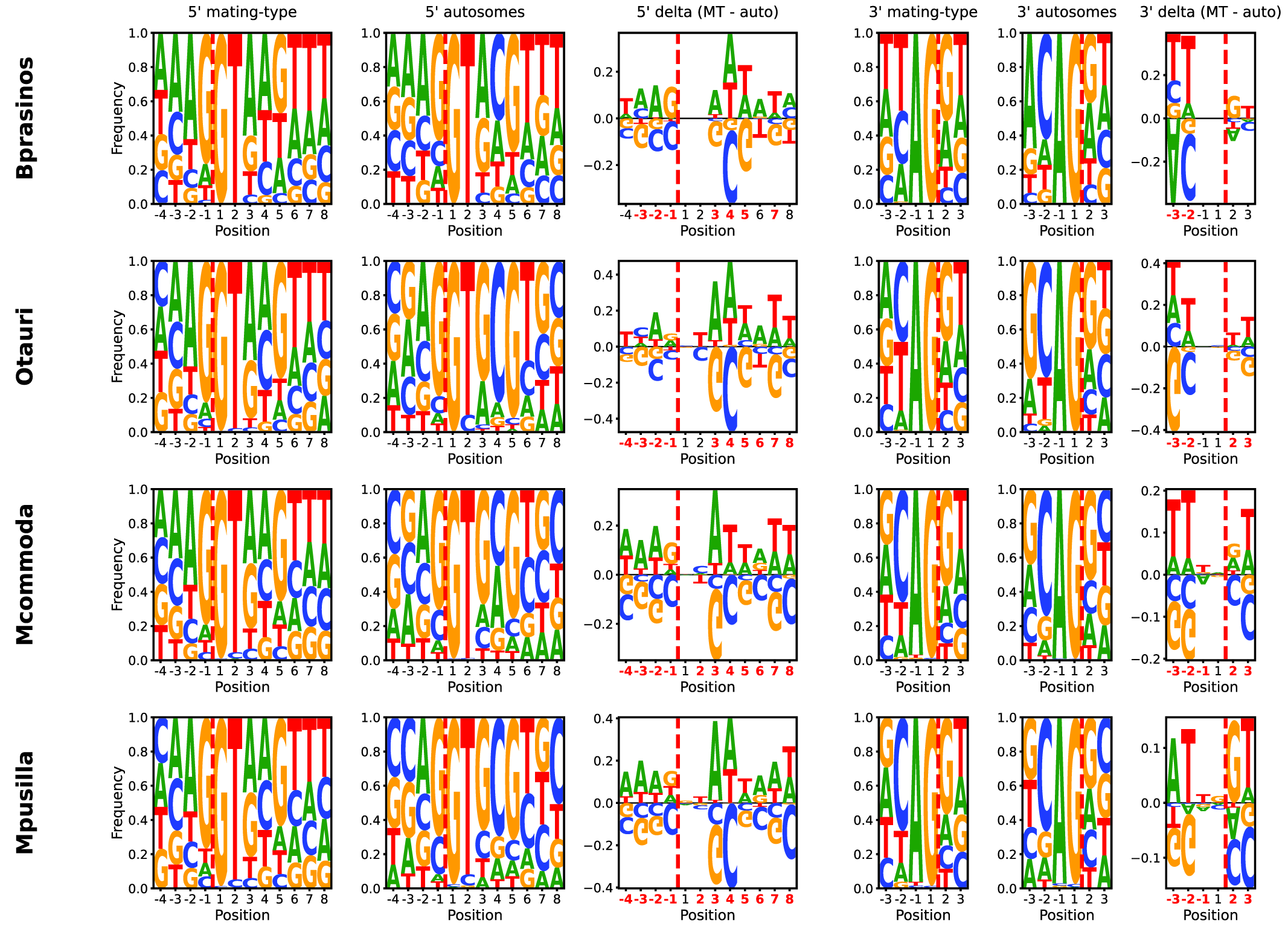

Supplement: S2 Fig — Data from M. pusilla is shown in Fig 2 and replicated here to facilitate comparison across species. Underlying data for this figure are available in the GitHub repository at https://github.com/russcd/mating-type-missplicing (file: data/{species}.features.branchpoints.tsv). (TIF) [file pbio.3003823.s002.tif]

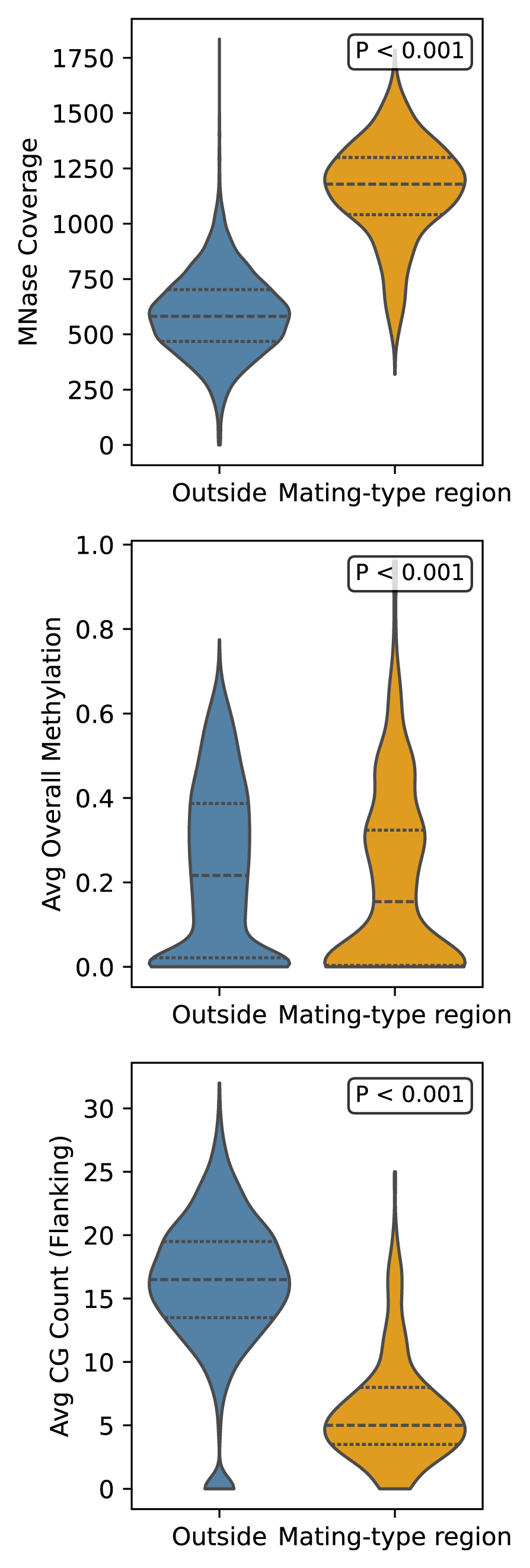

Supplement: S3 Fig — (Top) MNase-based library preparation coverage within introns. (Middle) Average per CG dinucleotide modification rates in bisulfite sequencing data. (Bottom) Number of CG dinucleotide sites in the 50 bp exons flanking each intron. We used a Mann–Whitney U test to determine p-values. MNase and methylation data from [22]. Underlying data for this figure are available in the GitHub repository at https://github.com/russcd/mating-type-missplicing (file: data/Mpusilla.features.methylation.tsv). (TIF) [file pbio.3003823.s003.tif]

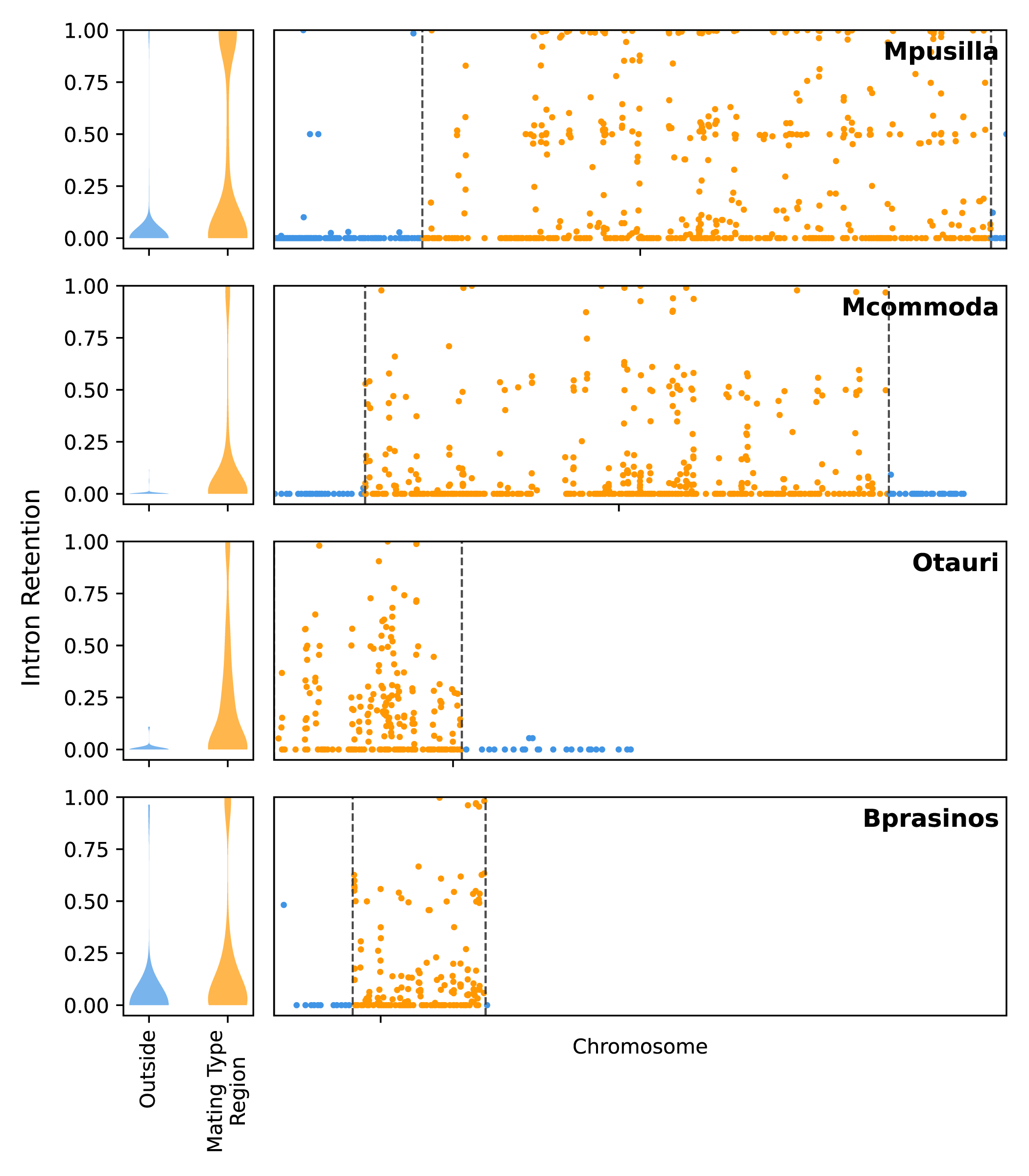

Supplement: S4 Fig — (Left), per intron retention frequency along autosomal regions (blue) and within the mating-type region (orange). (Right) Retention frequency for each intron plotted along each chromosome with colors as on the left panel. We obtained coordinates for mating type regions from [6]. Tick marks indicate the midpoint of chromosome in each genome assembly. Underlying data for this figure are available in the GitHub repository at https://github.com/russcd/mating-type-missplicing (file: data/{species}.features.branchpoints.tsv). (TIF) [file pbio.3003823.s004.tif]

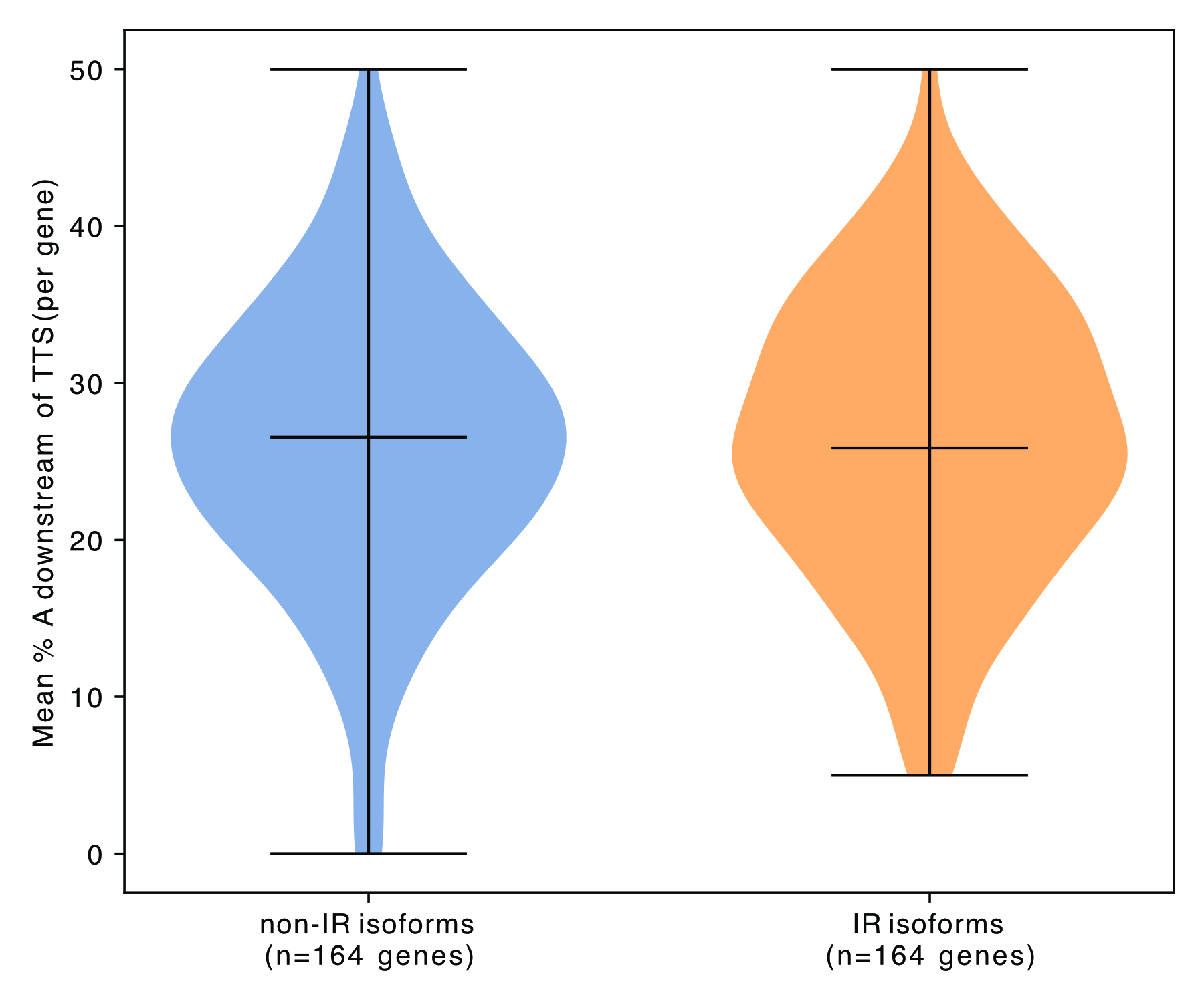

Supplement: S5 Fig — Each gene contributes one mean to the non-intron-retained violin (blue) and one to the intron-retained violin (orange). Underlying data for this figure are available in the GitHub repository at https://github.com/russcd/mating-type-missplicing (file: data/internal_priming_vs_IR_per_isoform.tsv). (TIFF) [file pbio.3003823.s005.tiff]
