# Supplementary material for: Splicing deficiency is driven by genomic erosion in non-recombining algal mating-type chromosomes
Source: PLoS Biol. 2026 Jun 25;24(6):e3003823. doi: 10.1371/journal.pbio.3003823 (PMC13298755; doi:10.1371/journal.pbio.3003823)
Supplement: S3 Table — ‘Present’ = exact 6/6 nucleotide match within the 50-nt branchpoint window upstream of the 3′ splice site. Ambiguity codes: V = A/C/G, R = A/G, S = C/G. Branchpoint is underlined. (DOCX) [file pbio.3003823.s008.docx]

|  |  | Mating-Type | | | Non-mating type | | |  |
| --- | --- | --- | --- | --- | --- | --- | --- | --- |
|  | Branchpoint Sequence | Present | Absent | Fraction | Present | Absent | Fraction | P-value |
| M. pusilla | CTGACS | 109 | 914 | 0.107 | 3180 | 4787 | 0.399 | 3.64E-215 |
| M. commoda | CTVACC | 81 | 860 | 0.086 | 1741 | 2744 | 0.388 | 8.11E-85 |
| O. tauri | ACTRAC | 50 | 385 | 0.115 | 1219 | 392 | 0.757 | 3.58E-136 |
| B. prasinos | RCTSAC | 22 | 308 | 0.067 | 527 | 637 | 0.453 | 6.22E-45 |
