# Supplementary material for: Splicing deficiency is driven by genomic erosion in non-recombining algal mating-type chromosomes
Source: PLoS Biol. 2026 Jun 25;24(6):e3003823. doi: 10.1371/journal.pbio.3003823 (PMC13298755; doi:10.1371/journal.pbio.3003823)
Supplement: S6 Table — Index corresponds to a random 8-nucleotide sequence unique to each replicate library. (DOCX) [file pbio.3003823.s011.docx]

| **Oligo ID** | **Sequence** |
| --- | --- |
| TSO-Smart-seq2 | AAGCAGTGGTATCAACGCAGAGTACATrGrGrG |
| Oligo_dT_Index1 | AAGCAGTGGTATCAACGCAGAGT [Index] ACTTTTTTTTTTTTTTTTTTTTTTTTTTTTTTVN |
| ISPCR primer | AAGCAGTGGTATCAACGCAGAGT |
| ISPCR_Anneal_Splint_1_F | ACTCTGCGTTGATACCACTGCTT  GCACGCACCGACAAACTCTGGCCGATTGTACTTTCTTATAAGGCGTAACACTAGACCATATCGTTTCTATAGATTAATTATGGCGAGAATTGCTAGCGAACTAGACAATTTTCGAAATAATCCTTTTTATAT AAGCAGTGGTATCAACGCAGAGT |
| ISPCR_Anneal_Splint_1_R | ACTCTGCGTTGATACCACTGCTT ATATAAAAAGGATTATTTCGAAAATTGTCTAGTTCGCTAGCAATTCTCGCCATAATTAATCTATAGAAACGATATGGTCTAGTGTTACGCCTTATAAGAAAGTACAATCGGCCAGAGTTTGTCGGTGCGTGC AAGCAGTGGTATCAACGCAGAGT |
