# Supplementary material for: Splicing deficiency is driven by genomic erosion in non-recombining algal mating-type chromosomes
Source: PLoS Biol. 2026 Jun 25;24(6):e3003823. doi: 10.1371/journal.pbio.3003823 (PMC13298755; doi:10.1371/journal.pbio.3003823)
Supplement: S7 Table — (DOCX) [file pbio.3003823.s012.docx]

| **NMD Protein** | **M. pusilla ortholog** | **M. commoda ortholog** | **B. prasinos ortholog** | **O. tauri ortholog** | **M. pusilla TPM** | **M. commoda TPM** | **B. prasinos TPM** | **O. tauri TPM** |
| --- | --- | --- | --- | --- | --- | --- | --- | --- |
| UPF1 | MICPUCDRAFT_56002 | MICPUN_65270 | Bathy10g01700 | OT_ostta09g02800 | 28.0 | 55.2 | 48.5 | 39.7 |
| UPF2 | MICPUCDRAFT_46957 | MICPUN_58110 | Bathy18g00500 | OT_ostta04g01650 | 31.8 | 51.0 | 25.9 | 101.8 |
| UPF3 | NA | NA | NA | OT_ostta15g01265 | NA | NA | NA | 17.7 |
