# Supplementary material for: Splicing deficiency is driven by genomic erosion in non-recombining algal mating-type chromosomes
Source: PLoS Biol. 2026 Jun 25;24(6):e3003823. doi: 10.1371/journal.pbio.3003823 (PMC13298755; doi:10.1371/journal.pbio.3003823)
Supplement: S8 Table — Here, we examined only genes with at least one intron, therefore requiring splicing for mature transcripts. (DOCX) [file pbio.3003823.s013.docx]

|  | MT Region | | Autosomal Regions | |  |
| --- | --- | --- | --- | --- | --- |
| Event | Total Events | Mean per gene | Total Events | Mean per gene | P-value Fisher’s Exact Test |
| A5SS | 41 | 0.12 | 36 | 0.01 | <0.0001 |
| A3SS | 57 | 0.17 | 59 | 0.02 | <0.0001 |
| ES | 31 | 0.09 | 97 | 0.04 | <0.0001 |
| RI | 629 | 1.83 | 1595 | 0.65 | <0.0001 |
