# Supplementary material for: Splicing deficiency is driven by genomic erosion in non-recombining algal mating-type chromosomes
Source: PLoS Biol. 2026 Jun 25;24(6):e3003823. doi: 10.1371/journal.pbio.3003823 (PMC13298755; doi:10.1371/journal.pbio.3003823)
Supplement: Attachment — Submitted filename: WeirdExpressionMatingTypeManuscript-ResponseDoc.pdf [file pbio.3003823.s014.pdf]

We thank both reviewers for their insightful and constructive feedback. We are pleased that both reviewers found the discovery of transcriptional degradation in mating-type chromosomes to be a novel and dramatic finding. In this revised version, we have significantly expanded the Introduction to provide better context for the *Mamiellales* system, added several control analyses on autosomal genes as requested, and clarified our figures and terminology.

REVIEWS:

Reviewer #1: This manuscript reports a discovery that is quite dramatic and unexpected. The authors show that in a group of green alga species, genes located in the region of the genome that determines mating type (called the UV region) produce mRNAs with a much higher rate of unspliced intron retention than genes in the rest of the genome, so that a large fraction of the transcripts from the UV region are unlikely to produce functional proteins. The difference between the two groups of genes is very pronounced (about 30-fold higher retention of introns) and it very clearly correlates with the location of the gene (Figure 1). The phenomenon is shared by 4 species of green algae (Mamiellales) that diverged >300 Mya, so it is a long-lived phenomenon.

The question then is what evolutionary factor(s) have caused this situation to arise? The authors propose, quite sensibly in my opinion, that it arises as a consequence of the fact that genetic recombination is suppressed in the UV region, so natural selection on these genes is inefficient, and they are also not subject to the same mutational processes (e.g. GC-biased gene conversion) as the rest of the genome. The authors show that, compared to the rest of the genome, genes in the UV regions have introns that are shorter, GC-poorer, and have weaker matches to consensus sequences around intron splice sites and branch sites. They also show that nucleosome occupancy and DNA methylation patterns are different between the UV region and the rest of the genome. They propose that these genomic changes are the result of the suppression of recombination in the UV region, and that the genomic changes have in turn led to a big increase in dysfunctional splicing. Nevertheless, the authors argue that the genes in the UV region are still functional: they still make a sufficient level of correctly spliced transcripts. They contrast this "transcriptional degradation" of the UV region with the degradation of genes on (for example) the mammalian Y chromosome, which has occurred mostly by pseudogenization or gene deletion.

The strong aspect of the manuscript is that the results it presents are, as far as I know, completely novel and unexpected, and it is a remarkable discovery. The manuscript has competent data analysis and discussion of the implications. I am aware of previous papers that reported that the UV regions of Mamiellales algae are very GC-poor, but not of ones that reported aberrant splicing in them, so I think this manuscript has high novelty.

The weak aspect of the manuscript is that the text is too brief. It does not provide sufficient background information for readers who are not already very familiar with Mamiellales genomes

and their mating type regions. The text, methods and figures/tables also sometimes provide insufficient detail about how some analyses were conducted.

The authors didn't provide line or page numbering. I've numbered the pages starting with the cover page as P1. I use § for paragraph numbers (including for parts of paragraphs, but excluding headers), and L for line number within a paragraph.

==

Major comments:

The Introduction fails to present some basic background information about the UV regions of Mamiellales algae:

- How large are these regions?
- How many genes do they contain?
- Is their gene density, and the number of introns per gene, the same as in the rest of the genome?
- How do we know where the ends of the non-recombining regions are?
- In each species, how similar is the U region to the V region? Are they completely different, or are there U/V paralogous gene pairs?
- Is it thought that all the genes in the UV regions participate in mating functions, or are there also housekeeping genes in these regions?
- Is the manuscript based on analysis of both the U and the V region from each species, or only one of these? Do we know whether U and V both show the same properties (GC-poorness, intron retention, etc)?

The authors examined 4 species of Mamiellales. Are the UV regions of these 4 species orthologous? The word chromosome (singular) in the manuscript's title implies that they are orthologous, as does the text at the end of P3 §3, but the text seem to doesn't cite any paper or data showing that they are orthologs. It is notable from Table S2 that the number of introns in the UV regions differs a lot among the species, so I guess that the number of genes also differs a lot, but the manuscript doesn't report this.

Thank you for pointing out this lack of background info in our introduction section. We have greatly expanded on the details of the UV regions (introduction Paragraphs 2 and 3) in the 4 Mamiellales species covered in this study. Included in these paragraphs are details about the size of the regions, their gene compositions and homology, their boundary definitions, the differences between MT+ and MT- alleles in *O. tauri*, the structural and dynamic characteristics of UV loci, and evidence for gene trafficking and translocations within UV loci in *O. tauri*. We have added additional citations (Benites et al. [ref7], Worden et al. [ref 6] Coelho et al. [ref 5] and Valiadi et al. [ref 8]) where they are relevant for the expanded introduction.

We hope this will provide better context regarding the structure and orthology of the UV regions to our readers.

Having read to the end of the Discussion, I am left with some unanswered questions.
- Why is there apparently no selection to relocate genes whose functions are unrelated to mating, from the UV region to autosomal regions where they would function better?

We think several factors explain gene persistence despite splicing deficiency. Our data show that MT genes produce substantial fractions of functional transcripts (Figure 3B). If 20-30% correct splicing satisfies fitness requirements, selection for mitigating factors is weaker. Successful translocations also require rare (and often deleterious) chromosomal events that preserve gene function while establishing new regulatory contexts. Additionally, unlike diploid sex chromosomes where redundancy permits gene loss, haploid systems require maintaining function in both mating types. Complete gene loss would immediately impact viability, while splicing deficiency producing 20-30% functional transcripts represents an evolutionary compromise that satisfies selective constraints while permitting molecular erosion.

- Or is there evidence that relocation has happened, e.g. some genes in the UV region of one species have autosomal orthologs in a different species?

Yes, comparative genomic analysis provides direct evidence that chromosomal translocations involving the MT region have occurred. Benites et al. [ref 7] identified gene families with members both inside and outside MT regions (46 genes in MT- and 30 genes in MT+ in O. tauri) have homologs located on diverse autosomes. We have included this additional information in introduction paragraph 3: "Comparative genomic analysis provides evidence for past translocations in O. tauri, where 46 gene families in MT- and 30 in MT+ have paralogs located on diverse autosomes (Benites et al. 2021)".

- Is there evidence that other gene degeneration processes, e.g. pseudogenes, are occurring in the UV regions?

There is evidence for other genomic degeneration processes beyond splicing deficiency. Only 23 of 216-619 genes are conserved across species [ref 7], indicating substantial loss or translocation compared to high autosomal retention. UV loci also show disrupted synteny, expanded repeat content, and decreased GC content [refs 6,7], hallmarks of recombination suppression and genomic decay. We have added these details to the expanded introduction as mentioned in the previous response.

==
Minor comments

P2 §1 (Abstract) L5: The text in many places describes mRNA isoforms as "truncated", but I'm not sure that this is the correct word, as opposed to "aberrant" or simply "intron-retaining". Looking at the example in Figure 3D, most of the transcript isoforms are roughly the same length as the top box, which I guess is the "correct" isoform, and some of them are actually longer because they retain introns.

Thank you for the suggestion, we have updated the text as “abberant isoforms” wherever it previously stated “truncated”.

Figure 1:

- Please provide a definition of "per intron retention frequency" (it's not defined in the Methods section either). Are the plotted points individual introns, or genes?

We have updated the Figure 1 text description so that it more accurately states the plotted points as individual introns, and not genes.

- It's difficult to see whether, on the chromosomes that contain the UV regions, the frequency of intron retention in the flanking non-UV regions is as low as in the rest of the genome. It would be helpful to comment on this and provide a zoomed-in view of those chromosomes.

We have added an additional supplementary figure (Figure S4) that provides a zoomed-in view of the chromosomes containing the MT region for each Mamiellales species. We hope this will provide a clearer visualization of the retention patterns in this region.

Figure 2A and Figure S2: It took me quite a while to realize that the base position called "the fifth position" in the text (P5 §1 L7) is the position labeled "4" in these figures. That's because the numbering in the figures includes a position zero. I suggest re-labeling the X-axis in the figures.

We have corrected Figure 2A and Figure S2 so that the x-axis is no longer zero-based, and the position numbers reflect the correct positions in the text.

P5 §1: The examples that are mentioned first in the text (i.e. the two exceptions on L3-4, and the G at the fifth position, L7) are quite subtle and difficult to see in Figure 2A/Fig S2. In contrast, the differences at the branch site (L12 and Table S3) are much more dramatic, so why not present them first?

Thank you for this suggestion. We agree that presenting the more dramatic branchpoint depletion first would improve clarity and impact. We have reordered results P2 to begin with branchpoint sequences, followed by splice-site flanking sequences, and then the subtle splice-site exceptions (“Introns in mating-type genes are highly divergent from those elsewhere in the genome. They are significantly shorter with lower GC content (Figure S1), both features that affect splicing efficiency [16,17]. Most strikingly,

branchpoint sequences which are critical for spliceosome assembly, are significantly depleted in all species within the mating-type region (p < 0.0001 for all species; Table S3)...".

P6 §3 L10: Does this text relate to the Evenness method (P13 §1 L1)? Are the results "data not shown", or is a supplementary table missing?

These two represent complementary measurements from different datasets. The gene-level variance on P6 measures covariation in intron retention across individual introns using short-read data and mixed-effects modeling. Evenness (P13) measures the distribution of full-length isoforms using R2C2 data. Both assess coordinated splicing within genes, but the short-read approach cannot resolve complete isoforms needed to calculate Evenness.

Figure 3 legend:
- Is this all in M. pusilla?

Yes, we have updated the text in Figure 3 to indicate that this is in M. pusilla.

- In A,B what are the boxes and whiskers? I'm puzzled by the result in panel B for 0-intron genes in non-UV regions - it seems to show a surprisingly large number of truncated CDSs but it has no box.

Here, the boxes represent interquartile ranges with median lines, while the whiskers extend to 1.5x the IQR. For 0-intron genes, in non-UV regions (panel B), all quartiles and the median all equal 1.0 (complete CDS retention), rendering the box invisible as a horizontal line at y=1.0. Points below 1.0 represent the outliers.

- In C, what do the thick and thin lines, and the different colors, represent? And the top box? I can guess, but please tell me.

Thank you for pointing out this omission. We have updated the Figure 3 legend to clarify these elements. The box at the top represents the canonical annotated gene structure. Each horizontal track below represents a distinct isoform, with colors assigned for visual distinction. Within each isoform, thick colored bars are exons, thin black lines are spliced introns,and thin medium-width lines are UTRs. This convention applies to both C and D.

P8 §2 L5 and Table S5:
- Does "80% of the length of the annotated CDS" take any account of the reading frame and translatability? It sounds like you are just considering any transcript that can be aligned at nucleotide level to >80% of the length of the canonical CDS to be functional.

Thank you for pointing out this need for clarification. Yes, our 80% threshold does account for reading frame and translatability. We specifically measure whether isoforms

retain at least 80% of the translatable coding sequence in the correct frame. Isoforms with frameshifts or premature stop codons that disrupt translation are classified as failing to meet this threshold even if they retain the nucleotide sequence. We have clarified this in the Results section (P8 §2: "We therefore indirectly estimated functionality by asking what fraction of full-length transcripts are derived from isoforms that contain at least 80% of the annotated CDS in the correct reading frame without premature termination").

- Wouldn't it be more pertinent to calculate the fraction of isoforms that can code for a protein >80% of the length of the canonical protein?

We agree that this is the appropriate metric, and this is what our analysis measures. We assess whether isoforms can produce proteins >= 80% the length of the canonical protein, accounting for proper reading frame and stop codon position.

- Table S5 has too many decimal places.

Thank you for this suggestion. We have reduced the max number of decimal places to 3.

- In Table S5, I don't understand the difference between columns 2-4 ("functional ontology terms") and columns 5-7 ("functional domains") and I didn't see any attempt to explain it. Do some domains have ontology terms attached, and others don't?

Thank you for requesting clarification. InterProScan annotates proteins using two complementary approaches: (1) functional domains are structural/sequence features identified by databases like Pfam, SMART, and PROSITE and (2) functional ontology terms are Gene Ontology (GO) annotations describing biological process, molecular function, or cellular component. These annotations are not one-to-one: some domains have associated GO terms while others do not, and some GO terms can be assigned based on sequence similarity without requiring a characterized domain. We analyzed both metrics because they capture different aspects of protein function—domains reflect structural integrity while GO terms reflect predicted biological roles. We have added this explanation to the Table S5 legend.

- The description on P8 §2 L11-15 of an example result from this analysis compares the domain content of *isoforms* from autosomal versus UV regions. Do you really mean isoforms here, rather than transcripts? In other words, would each isoform in Fig 3D be weighted equally, and not by the number of times you detected it in the transcript pool?

Yes, this is correct as interpreted. Specifically, we are measuring isoforms without weighting by their transcript abundance. This is because R2C2 excels at identifying the complete set of full-length isoforms by generating consensus sequences, but does not provide reliable quantification of the isoform abundances. Our analysis thus assesses what qualitative fraction of a gene's transcript isoform set retains functional capacity.

P8 §3 L11: Are intergenic distances also systematically shortened?

This is correct. We have expanded the introduction to state that the gene density in the UV regions are systematically less than in the autosomal regions ("Like non-recombining sex chromosomes, UV loci show disrupted synteny, lower gene density, expanded repeat content, decreased GC nucleotide content (Worden et al. 2009; Benites et al. 2021; Coelho et al. 2018), and more open chromatin [8] compared to autosomes.").

Table S1: Please add a column showing the ratio between the mean retention level in autosomes versus mating-type regions.

We have updated Table S1 to include the ratio of mean retention level in mating-type vs. autosomal regions.

For both Table S2 and Table S3, I'm not as good at mental arithmetic as the authors are, so please add columns showing the frequencies (e.g. in Table S2, in M. pusila, G at the 5th position is at frequency 0.64 in mating-type genes and frequency 0.71 in the non-mating type genes). This would make it easier to see the data supporting the statements in the text at P5 §1.

Thank you for the suggestion, we have updated Tables S2 and S3 to include fractions and highlight how dramatic the branchpoint deletions are.

Table S3: Does "present" indicate that a perfect (6 out of 6) match to the branchpoint sequence is present, or what? Please add a footnote clarifying this. And is the branchpoint sequence shown the consensus sequence from all introns in the genome?

Yes, "present" does indicate an exact (6/6 nucleotide) match to the branchpoint sequence motif located anywhere in the 50-nt window upstream of the 3' splice site. The branchpoint sequences shown were identified by DREME motif discovery on branchpoint windows extracted from all annotated introns in each genome. We've added a footnote to Table S3 clarifying these details.

P6 §2 L2 and Figure S3: It's unclear whether the experimental methylation and MNase coverage data comes from a previous publication by another group (ref. 8, cited on P6 §2 L2) or is new data from this study (P11 §5 mentions that chromatin analysis was done for this study).

Thank you for catching this ambiguity. The MNase coverage and methylation data are from a previous publication (Huff & Zilberman 2014, reference [25]), not new data generated for this study. Reference [8] (Valiadi et al. 2025) provides independent chromatin accessibility evidence supporting altered chromatin structure in MT regions. We have added citation [25] to both the Results text ("MNase-based measurement of chromatin accessibility (Huff and Zilberman 2014) suggests") and Figure S3 ("MNase and methylation data from (Huff and Zilberman 2014).

“) to clarify the data source.

Figure 4 is not cited anywhere in the text. It looks more like a graphical abstract.

Thank you for catching this omission. Figure 4 is indeed intended as a graphical summary of our proposed model. We have added a citation to it in the Discussion section where we describe the cascade of molecular changes leading to splicing deficiency (“We propose that recombination suppression triggered a cascade of interconnected molecular changes and distinct selective pressures that collectively explain these widespread transcript-level defects (Figure 4).”).

Supplementary Tables and Figures: Please don't put page breaks in the middle of tables, or between a figure and its legend.

We have gone through and ensured there are no page breaks in any of the tables, or between a figure and its legend.

P2 §1 (Abstract) L6: By "This suggests…" do you mean "This preservation suggests…"?

Yes, this is correct. The text has been updated to reflect this (“This preservation suggests that splicing defects…”).

P3 §2 L5: Would "genetic decay" not be a better description than "genomic decay"?

This is true, we have edited at P3 §2 L5 from “genetic decay” to “genomic decay”.

P5 §1 L2 citation should be to Figure S2.

Thank you for catching this error. We have corrected the citation from Figure S1 to Figure S2 for the sentence discussing canonical splice site frequencies.

P9 §1 should begin "We propose that this collective action…" to make it clear that this paragraph is a continuation of the hypothesis.

We have updated the text here as suggested to make this sentence more clear that it is a continuation of our hypothesis (“We propose that this collective action of mutational biases…”).

P9 §1 L8: Please make it clearer that ref. 12 is a paper about transcription in human genes, not green algal mating type locus genes.

We have highlighted in our citation that this paper is regarding transcription in human genes (“Third, altered chromatin structure appears to elevate overall gene expression levels in human [12]”).

Reviewer #2: The authors perform a comparative transcriptomics analysis across species and genomic region types (mating-type and autosomes), using both publicly available short read RNA sequencing data and long read RNA sequencing that they generate themselves. The first and perhaps most striking result is that intron retention frequencies are considerably higher in mating-type regions than outside (Figure 1), in all 4 species. They analyze frequencies of splicing-regulated motifs and identify several differences between the two categories of genes (including a depletion in canonical branchpoint sequences), suggesting a potential mechanistic basis for the differences observed in intron retention frequencies. Interestingly, intron length and GC content (both of which are lower in mating-type genes) seem to have high predictive power for intron retention frequencies. Both these features are strongly associated with the reduction in recombination rate. Finally, long-read RNA
sequencing analysis showed that isoforms in mating type regions are often truncated. All these observations indicate that the increased intron retention rate in mating type regions is due to less efficient splicing, likely through an accumulation of slightly deleterious mutations that cannot be efficiently purged due to reduced recombination.

Overall, I find the manuscript very convincing. I appreciate the fact that the analyses are simple and to the point. The intron retention results are quite striking. I have some suggestions below but overall I am confident that this manuscript is ultimately worthy of publication.

We thank the reviewer for their kind words.

Major comments:

- I'm not sure why the random forest analysis was not performed on autosomal genes. There is definitely lower variation in intron retention rates for this class of genes, but since there are many more genes, a classification could still be interesting. It would be good to know whether the relationships between intron retention on one hand, and GC-content, intron length, expression level etc. are the same for the two categories of genes.

We appreciate the suggestion to use the autosomal genes as a comparative model. We did perform a Random Forest analysis on autosomal genes to test whether the same features predict intron retention events in autosomal regions. Only 0.6%-2.6% of introns show retention above our threshold across species. This creates severe class imbalance that prevents meaningful model training. Autosomal models achieved F1 scores of only 0.00-0.17 compared to 0.65-0.81 for mating-type models. The models essentially learn to predict “not retained” for all autosomal introns because retention events are too rare. We have added the results from this analysis in Table S4, and updated the legend accordingly. Additionally, we have updated the Results (“In contrast, Random Forest models trained on autosomal introns failed to achieve meaningful predictive performance (F1=0.00-0.17) due to extreme class imbalance where retained introns comprise only 0.6-2.6% of autosomal introns (Table S4).”) and Methods (“We also trained Random Forest models on autosomal introns using the same procedure. The extreme rarity of

retention events in autosomal regions prevented effective model training, yielding low F1 scores (Table S4). ") sections to include this analysis.

- I have some trouble with the observation that gene expression levels are positively correlated with intron retention rates, across genes in mating type regions. In previous studies performed for a wide variety of species (Saudemont et al, 2018; Bénitière et al, 2024), the opposite correlation between alternative splicing rates and gene expression levels are observed in the great majority of species. Saudemont et al specifically showed that intron retention rates are negatively correlated with gene expression levels in humans and Paramecium. This was explained by the proposal of an increased cost of splicing errors in highly expressed genes. Why would the correlation go the other way around these algal species? I am not an expert in recombination suppresion around mating type loci, so this may be naïve: Could the expression level be anticorrelated with the rate of (historical) recombination in these regions? It would be interesting to perform the same analysis across
autosomal genes: can we observe a similar positive correlation between intron retention rates and expression levels?

Thank you for pointing out this important deviation from the trends reported by Saudemont et al. (2017) and Bénitière et al. (2024). You are correct that our observation appears contradictory to previous work showing that selection minimizes splicing errors in highly expressed genes.

As we expanded in the introduction, the UV region is highly distinct from the remainder of the genome with a >20% shift in GC content and approximately 2x the MNase coverage on average. Our hypothesis is that the region is so fundamentally distinct, perhaps on par with Y chromosomes which maintain different chromatin but do not preserve gene fidelity in the face of genomic erosion, that an equivalent set of circumstances may not exist across the human or most eukaryotic genomes. Specifically, we propose that the positive correlation between missplicing and expression reflects a chromatin-mediated structural difference in non-recombining regions. This altered chromatin landscape accelerates RNA polymerase II transcription kinetics, and in expressed genes, the combination of rapid Pol II elongation and high transcriptional flux outpaces co-transcriptional spliceosome assembly, leading to increased retention. Furthermore, inefficient selection in the non-recombining region cannot correct this chromatin-level defect.

There is also generally a much weaker positive correlation between intron retention and gene expression in autosomal genes (Spearman's ρ = 0.06-0.12 across species). This difference between genomic regions supports our interpretation that the positive correlation is specific to the altered chromatin environment of the mating-type region, not a general feature of these species. We have expanded the Discussion (paragraph 2, lines 10-16) to clarify this chromatin-transcription-splicing mechanistic link and added the autosomal comparison.

- Likewise, it would be interesting to test whether the number of introns is associated with intron retention rates in these species, for mating type genes and for autosomal genes. Figure 3 shows the relationship between the number of introns and the proportion of truncated isoforms, but the two observations may be independent.

We thank the reviewer for this suggestion. We tested the association between intron number and retention rate as suggested. In UV loci genes, we observed a strong positive correlation (Spearman's ρ = 0.6 across species, p< 0.0001 for all), while autosomal genes showed weak to negligible correlations (Spearman's ρ = 0.06-0.22 across species, p<0.0001 for all). This pattern is consistent with our hypothesis that mating-type region-specific factors (weakened splice sites, altered chromatin) systematically impair splicing efficiency, with effects compounding in multi-intron genes.

We have added this result to the Discussion paragraph 2 ("gene expression and intron retention are weakly positively correlated even in autosomal regions, where selection can operate efficiently to minimize intron retention (Spearman's ρ = 0.06-0.12 across species, p <0.0001).").

- What is known about nonsense-mediated decay in these species? I ask this because it seems that properties such as frameshifts and presence of stop codons do not appear to correlate with intron retention rates across mating type genes. It would be good to analyze the rela

Thank you for this question. Nonsense-mediated decay (NMD) has not been extensively studied in Mamiellales, but we identify core NMD machinery components (UPF1 and UPF2) encoded in all four genomes and expressed at moderate to high levels in our RNA-seq data (see table below), suggesting the pathway is functional. We have added this table to the main text as Table S7 and updated P6 §2 to include this result.

NMD Protein	M. pusilla ortholog	M. commoda ortholog	B. prasinos ortholog	O. tauri ortholog	M. pusilla TPM	M. commoda TPM	B. prasinos TPM	O. tauri TPM
UPF1	MICPUCDRAFT_56002	MICPUN_65270	Bathy10g01700	OT_ostta09g02800	28.0	55.2	48.5	39.7
UPF2	MICPUCDRAFT_46957	MICPUN_58110	Bathy18g00500	OT_ostta04g01650	31.8	51.0	25.9	101.8
UPF3	NA	NA	NA	OT_ostta15g01	NA	NA	NA	17.7

				265				

- How can one explain the relationship between intron retention rate and position with respect to the gene TSS? If I interpret the graph in Figure 2 correctly (of which I am not sure, cf. comment below), it seems that introns in 3' end of the gene are more often retained. Does this observation hold with long-read sequencing data? Short-read sequencing with polyA selection induces a representation bias towards the end of the transcript, which could induce an estimation bias - retained introns can be more readily detected if the overall coverage is high.

Thank you for this important methodological concern. We performed a coverage analysis along gene bodies across all four species. Only O. tauri shows slight 3' enrichment (1.15-fold), yet all four species exhibit the TSS-distance effect (Figure 2E), which makes estimation bias unlikely. Our long-read isoform data qualitatively support this pattern: we observe numerous isoforms with 3' intron retention while 5' introns are spliced in the same transcripts (Figure 3D), confirming these events are not coverage artifacts (though R2C2 cannot quantify retention rates).

Additionally, rMATS calculates retention as a ratio (inclusion reads / [inclusion + exclusion reads]) at each junction, so absolute coverage differences do not directly bias estimates. Random Forest models control for expression (which correlates with coverage), yet TSS-distance remains predictive. The pattern is also consistent with co-transcriptional splicing models where downstream introns are spliced less efficiently as RNA polymerase II accelerates through altered UV loci chromatin.

- Finally, do the observations hold for other types of alternative splicing events? One easy way to estimate this is with the alternative variant ratio proposed by Bénitière et al 2024, based on spliced reads. Otherwise one could directly assess exon skipping rates for example.

Thank you for this suggestion. We focused on intron retention (RI) because the majority of other rMATS event calls are redundant with RI. We confirmed this by checking the overlap of other AS events with RI events: 50–85% of skipped exon (SE) events, 66–71% of alternative 3' splice site (A3SS) events, and 38–50% of alternative 5' splice site (A5SS) events have coordinates overlapping an RI call in the same gene. This redundancy arises because rMATS independently tests each event type using distinct read configurations without enforcing exclusivity at that location. Consequently, reads supporting intron retention are also classified as alternative splice sites or skipped exons.

We do discuss elevated A3SS and A5SS rates in the mating-type region (P5 §1 and Figure 2A), which aligns with our model that weakened splice site recognition drives multiple forms of splicing dysfunction. The comprehensive picture from long-read isoforms (Figure 3) further demonstrates that diverse aberrant splicing patterns in addition to RI are present in the isoform sets .

Minor comments:

- It would be good to show a distribution of gene expression levels within and outside mating type regions in a supplementary figure, to support the analysis on methylation rates.

Thank you for this suggestion. Elevated gene expression in the MT region has been observed in previous publications, and we have added this to our expanded Introduction, along with the relevant citations (P3 line 3: "Notably, UV loci in *Mamiellales* also show higher gene expression than the autosomal regions (Worden et al. 2009; Valiadi M, Harrison K, Loe-Mie Y, Wil...).").

- In figure 2, B-E, it is not clear how the "partial dependence" on the Y axis should be interpreted. A couple of phrases helping with the interpretation would be helpful.

Thank you for this suggestion. We have added explanatory text to the Figure 2 legend clarifying that partial dependence plots show the marginal effect of each feature on predicted intron retention probability while averaging over all other features in the model.

- This paragraph is not clear:
"Additionally, genes within the mating type region express all isoforms as
a more similar fractions of the total transcript pool than genes in autosomal regions" - do you mean that isoform frequency is less skewed towards one major isoform? This could be clarified.

This is correct, we do mean isoform frequency is less skewed towards a major isoform in mating-type genes. In other words, mating-type genes distribute their transcript output more evenly across multiple isoforms rather than producing one major isoform with rare alternatives. We have revised this sentence for clarity at P6 §3 ("Additionally, genes within the mating-type region show more evenly distributed isoform expression, with less dominance by a single major isoform, compared to autosomal genes ($p < 0.0001$, GLM; see Methods).").
