# Supplementary material for: Splicing deficiency is driven by genomic erosion in non-recombining algal mating-type chromosomes
Source: PLoS Biol. 2026 Jun 25;24(6):e3003823. doi: 10.1371/journal.pbio.3003823 (PMC13298755; doi:10.1371/journal.pbio.3003823)
Supplement: Attachment — Submitted filename: Mating Type Manuscript Rev2 Response Doc.docx [file pbio.3003823.s015.docx]

REVIEWS:

Reviewer #2: I would like to thank the authors for their answer to my comments. While the manuscript has been improved by the revision, I still have some concerns regarding the manuscript.

1) My suggestion regarding the analysis of other types of alternative splicing events was not addressed. I understand that rMATS cannot properly distinguish intron retention and other AS events, at least in this context. However, one could in fact use other software (or just simple home-made scripts) to analyze alternative splice site usage. Alternative splice sites are detected using exclusively spliced reads. Thus, their detection cannot be affected by intron retention events. This can be easily done with home-made scripts, by analyzing intron coordinates from mapped reads (read mapping software such as HISAT2 can output the coordinates of detected introns).

**We thank the reviewer for this helpful suggestion and we apologize for not properly addressing it in the first round of edits. We implemented the analysis as proposed, using junction coordinates extracted directly from our full-length R2C2 isoform consensus sequences. We focus on the R2C2 data because it is the most straightforward to characterize for the full range of possible splicing events as suggested by the reviewer. For each gene with at least two isoforms, we counted all unique exon junctions and quantified the number of alternative 5' splice sites (A5SS, alternative donors), alternative 3' splice sites (A3SS, alternative acceptors), skipped exons (SE), and retained introns. Full per-region counts are provided in Supplementary Table S8 and included below to facilitate review.**

**Classical alternative splicing events (A5SS, A3SS, and SE) are rare in this dataset. Together, they account for only ~100 events in the mating-type region and ~190 events across all 2,458 multi-isoform autosomal genes, and are 6.3 times fewer (629 retained introns) than in mating-type, and 8.4 times fewer (1,595 retained introns) than in autosomal. This confirms that intron retention is the dominant class of alternative splicing in *M. pusilla*. Nonetheless, each AS event category is enriched on a per-gene basis in the mating type region relative to autosomes (A5SS 8.2×, A3SS 6.9×, SE 2.3×, and RI 2.8×; p < 0.0001 for each comparison) indicating that whatever mechanism elevates splicing variability in the MT region relative to the autosomes operates broadly across event types, not only on intron retention. Taken together, these results both address the reviewer's request to quantify non-IR AS events and strengthen the manuscript's conclusion that the MT region harbors an unusual splicing environment.**

Table S8: Alternative splicing event quantification from R2C2 long reads within the MT and autosomal regions in *M. pusilla.* Here we examined only genes with at least one intron, therefore requiring splicing for mature transcripts.

|  | MT Region | | Autosomal Regions | |  |
| --- | --- | --- | --- | --- | --- |
| Event | Total Events | Mean per gene | Total Events | Mean per gene | P-value Fisher’s Exact Test |
| A5SS | 41 | 0.12 | 36 | 0.01 | <0.0001 |
| A3SS | 57 | 0.17 | 59 | 0.02 | <0.0001 |
| ES | 31 | 0.09 | 97 | 0.04 | <0.0001 |
| RI | 629 | 1.83 | 1595 | 0.65 | <0.0001 |

2) The reason I insist on looking at additional types of alternative splicing events is that, upon rereading the manuscript, I am worried about a potentially important confounding factor: namely, the presence of internal priming due to the presence of AT-rich sequences. The authors note that there are significantly more alternative termination sites in the mating type region. According to their own interpretation, this could be due to mis-priming of oligo-dT primers during library preparation. But this could in fact also explain much of the observed "intron retention". If mis-priming of oligo-dT priming has occurred, it is likely that the sequenced transcriptome contains a non-negligible fraction of transcripts that lack a polyA tail. If a high fraction of the transcripts captured by RNA sequencing in the mating type region are immature, this would lead to an artificial increase in the estimation of intron retention rates. This could also explain the puzzling observation that most retained introns appear towards the 3' end of the transcripts - I don't understand why genuine intron retention would be 3' biased, while inclusion of immature transcripts would indeed generate apparent retained introns in the 3' end of the transcripts. With long read sequencing, one could check whether intron retention is also observed at high frequencies when considering only reads that contain a proper polyA tail. I apologize for not realizing this major issue at the first round of review.

**We thank the reviewer for raising this important concern and for their clear explanation of the potential problem. oligo-dT mispriming on genomic A-rich stretches is a possible important artifact class. Although we do indicate that mispriming is a possible contributor to 3’ TSS variation in the text, we included this consideration out of an abundance of caution. The *M. pusilla* mating-type region has an overall GC% similar to the human genome (41% in human vs 42% *M. pusilla*), and we therefore think mispriming would actually be quite uncommon in our data *a priori*.**

**We are able to address this concern directly using our Oxford Nanopore R2C2 long-read data, which enables us to statistically evaluate each full-length isoform for missplicing artifacts. After doing so, we find that internal priming is unlikely to account for the elevated intron retention we observe in the MT region.**

**Our R2C2 isoforms were assembled with Mandalorion v4.6.0 using Acutoff = 0.5, which discards any read whose 20 bp downstream genomic window exceeds 50% A, serving as a read-level internal priming filter. Additionally, we filtered out any isoforms that SQANTI3 flagged as putative RT-switching artifacts. These are the default parameters of each software package and are thought to help mitigate the impact of mispriming.**

**As a further test of this concern, we analyzed the MT region by fitting a logistic regression model of intron retention status on downstream A content in 20 bp windows from the apparent transcription stop site. Our model, which has the form P(has_IR) ~ percent_A + gene_id, included a gene-level categorical fixed effect to control for the gene-level variation in intron retention propensity. This tests whether an isoform’s downstream A content predicts intron retention relative to other isoforms of the same gene. We restricted this model to the 164 genes in the MT region with at least one IR isoform and one non-IR isoform (n = 1,393 isoforms). We find that the percent of A nucleotides adjacent to the transcription termination site has no predictive power for intron retention rates (standard error = 0.0035 and p = 0.31, now Figure S5 and see below). This indicates that mispriming of nascent transcripts is unlikely to drive the effects we report here. Additionally, as the reviewer emphasizes above, other missplicing events besides intron retention are also elevated within isoforms derived from genes in the mating type region, consistent with incorrectly spliced mature transcripts.**

**While we don’t understand the molecular basis of the observed bias for intron retention closer to the 3’ end of the transcript, we suspect that this pattern is likely due to an initial proper co-transcriptional splicing coordination which degrades (due to reasons proposed in our model; Fig. 4) partway through transcription, thus rendering it more prone to intron retention towards the 3’ end of the transcript and early RNA Pol II termination. Previous work showed that upstream introns are more often spliced before downstream ones in *Drosophila*** [**(Drexler et al. 2020)**](https://paperpile.com/c/9Wf1Lz/DSwh)**.**

**Figure S5:** Per-gene mean percent A downstream of the TTS (20 bp window) for the 164 mating-type genes that contain both intron-retained and non-intron-retained isoforms. Each gene contributes one mean to the non-intron-retained violin (blue) and one to the intron-retained violin (orange).
