# Supplementary material for: Splicing deficiency is driven by genomic erosion in non-recombining algal mating-type chromosomes
Source: PLoS Biol. 2026 Jun 25;24(6):e3003823. doi: 10.1371/journal.pbio.3003823 (PMC13298755; doi:10.1371/journal.pbio.3003823)
Supplement: Peer review history

# Editor report: Decision Letter 0

**DOI:** 10.1371/journal.pbio.3003823.r001

**Authors:** Richard Hodge

11 Dec 2025

Dear Dr Condon,

Thank you for submitting your manuscript entitled "Extensive Splicing Deficiency in a Degenerating Mating-Type Chromosome" for consideration as a Research Article by PLOS Biology. Please accept my apologies for the delay in getting back to you this week as we consulted with an academic editor about your submission.

Your manuscript has now been evaluated by the PLOS Biology editorial staff, as well as by an academic editor with relevant expertise, and I am writing to let you know that we would like to send your submission out for external peer review.

IMPORTANT: After discussions with the rest of the editorial team, we think that your manuscript would be a better fit as a Short Report at the journal given that the manuscript has 4 main figures (https://journals.plos.org/plosbiology/s/what-we-publish#loc-short-reports). Upon resubmission (see details below), I would be grateful if you could please tick 'Short Report' as the article type in the drop down menu in the online submission form.

Before we can send your manuscript to reviewers, we need you to complete your submission by providing the metadata that is required for full assessment. To this end, please login to Editorial Manager where you will find the paper in the 'Submissions Needing Revisions' folder on your homepage. Please click 'Revise Submission' from the Action Links and complete all additional questions in the submission questionnaire.

Once your full submission is complete, your paper will undergo a series of checks in preparation for peer review. After your manuscript has passed the checks it will be sent out for review. To provide the metadata for your submission, please Login to Editorial Manager (https://www.editorialmanager.com/pbiology) within two working days, i.e. by Dec 13 2025 11:59PM.

If your manuscript has been previously peer-reviewed at another journal, PLOS Biology is willing to work with those reviews in order to avoid re-starting the process. Submission of the previous reviews is entirely optional and our ability to use them effectively will depend on the willingness of the previous journal to confirm the content of the reports and share the reviewer identities. Please note that we reserve the right to invite additional reviewers if we consider that additional/independent reviewers are needed, although we aim to avoid this as far as possible. In our experience, working with previous reviews does save time.

If you would like us to consider previous reviewer reports, please edit your cover letter to let us know and include the name of the journal where the work was previously considered and the manuscript ID it was given. In addition, please upload a response to the reviews as a 'Prior Peer Review' file type, which should include the reports in full and a point-by-point reply detailing how you have or plan to address the reviewers' concerns.

During the process of completing your manuscript submission, you will be invited to opt-in to posting your pre-review manuscript as a bioRxiv preprint. Visit http://journals.plos.org/plosbiology/s/preprints for full details. If you consent to posting your current manuscript as a preprint, please upload a single Preprint PDF.

Feel free to email us at plosbiology@plos.org if you have any queries relating to your submission.

Kind regards,

Richard

Richard Hodge, PhD

Senior Editor, PLOS Biology

rhodge@plos.org

PLOS

Empowering researchers to transform science

Carlyle House, Carlyle Road, Cambridge, CB4 3DN, United Kingdom

California (U.S.) corporation #C2354500, based in San Francisco

# Peer review: Decision Letter 1

**DOI:** 10.1371/journal.pbio.3003823.r002

**Authors:** Richard Hodge

3 Feb 2026

Dear Dr Condon,

Thank you for your patience while your manuscript "Extensive Splicing Deficiency in a Degenerating Mating-Type Chromosome" went through peer-review at PLOS Biology. Please accept my sincere apologies for the delays that you have experienced during the peer review process. Your manuscript has now been evaluated by the PLOS Biology editors, an Academic Editor with relevant expertise, and by two independent reviewers.

In light of the reviews, which you will find at the end of this email, we are pleased to offer you the opportunity to address the comments from the reviewers in a revision that we anticipate should not take you very long. Specifically, Reviewer #1 notes that the manuscript is too brief and requests additional background information in the Introduction and reporting details. In addition, Reviewer #2 highlights the observation that the most expressed genes have the most diverse splice forms, opposite to that seen by Benitiere et al. We ask that this is discussed further in the text and wonder if a different metric is required such as splice forms per transcript.

We will then assess your revised manuscript and your response to the reviewers' comments with our Academic Editor aiming to avoid further rounds of peer-review, although we might need to consult with the reviewers, depending on the nature of the revisions.

In addition to these revisions, you may need to complete some formatting changes, which you will receive in a follow up email. A member of our team will be in touch with a set of requests shortly. If you do not receive a separate email within a few days, please assume that checks have been completed, and no additional changes are required.

We expect to receive your revised manuscript within 1 month. Please email us (plosbiology@plos.org) if you have any questions or concerns, or would like to request an extension.

At this stage, your manuscript remains formally under active consideration at our journal; please notify us by email if you do not intend to submit a revision so that we withdraw the manuscript.

**IMPORTANT - SUBMITTING YOUR REVISION**

Your revisions should address the specific points made by each reviewer. Please submit the following files along with your revised manuscript:

1. A 'Response to Reviewers' file - this should detail your responses to the editorial requests, present a point-by-point response to all of the reviewers' comments, and indicate the changes made to the manuscript.

*NOTE: In your point-by-point response to the reviewers, please provide the full context of each review. Do not selectively quote paragraphs or sentences to reply to. The entire set of reviewer comments should be present in full and each specific point should be responded to individually.

You should also cite any additional relevant literature that has been published since the original submission and mention any additional citations in your response.

2. In addition to a clean copy of the manuscript, please also upload a 'track-changes' version of your manuscript that specifies the edits made. This should be uploaded as a "Revised Article with Changes Highlighted " file type.

*Resubmission Checklist*

When you are ready to resubmit your revised manuscript, please refer to this resubmission checklist: https://plos.io/Biology_Checklist

To submit a revised version of your manuscript, please go to https://www.editorialmanager.com/pbiology/ and log in as an Author. Click the link labelled 'Submissions Needing Revision' where you will find your submission record.

Please make sure to read the following important policies and guidelines while preparing your revision:

*Published Peer Review*

Please note while forming your response, if your article is accepted, you may have the opportunity to make the peer review history publicly available. The record will include editor decision letters (with reviews) and your responses to reviewer comments. If eligible, we will contact you to opt in or out. Please see here for more details:

https://blogs.plos.org/plos/2019/05/plos-journals-now-open-for-published-peer-review/

*PLOS Data Policy*

Please note that as a condition of publication PLOS' data policy (http://journals.plos.org/plosbiology/s/data-availability) requires that you make available all data used to draw the conclusions arrived at in your manuscript. If you have not already done so, you must include any data used in your manuscript either in appropriate repositories, within the body of the manuscript, or as supporting information (N.B. this includes any numerical values that were used to generate graphs, histograms etc.). For an example see here: http://www.plosbiology.org/article/info%3Adoi%2F10.1371%2Fjournal.pbio.1001908#s5

*Blot and Gel Data Policy*

We require the original, uncropped and minimally adjusted images supporting all blot and gel results reported in an article's figures or Supporting Information files. We will require these files before a manuscript can be accepted so please prepare them now, if you have not already uploaded them. Please carefully read our guidelines for how to prepare and upload this data: https://journals.plos.org/plosbiology/s/figures#loc-blot-and-gel-reporting-requirements

*Protocols deposition*

To enhance the reproducibility of your results, we recommend that if applicable you deposit your laboratory protocols in protocols.io, where a protocol can be assigned its own identifier (DOI) such that it can be cited independently in the future. Additionally, PLOS ONE offers an option for publishing peer-reviewed Lab Protocol articles, which describe protocols hosted on protocols.io. Read more information on sharing protocols at https://plos.org/protocols?utm_medium=editorial-email&utm_source=authorletters&utm_campaign=protocols

Thank you again for your submission to our journal. We hope that our editorial process has been constructive thus far, and we welcome your feedback at any time. Please don't hesitate to contact us if you have any questions or comments.

Best regards,

Richard

Richard Hodge, PhD

Senior Editor, PLOS Biology

rhodge@plos.org

----------------------------------------------------------------

REVIEWS:

Reviewer #1: This manuscript reports a discovery that is quite dramatic and unexpected. The authors show that in a group of green alga species, genes located in the region of the genome that determines mating type (called the UV region) produce mRNAs with a much higher rate of unspliced intron retention than genes in the rest of the genome, so that a large fraction of the transcripts from the UV region are unlikely to produce functional proteins. The difference between the two groups of genes is very pronounced (about 30-fold higher retention of introns) and it very clearly correlates with the location of the gene (Figure 1). The phenomenon is shared by 4 species of green algae (Mamiellales) that diverged >300 Mya, so it is a long-lived phenomenon.

The question then is what evolutionary factor(s) have caused this situation to arise? The authors propose, quite sensibly in my opinion, that it arises as a consequence of the fact that genetic recombination is suppressed in the UV region, so natural selection on these genes is inefficient, and they are also not subject to the same mutational processes (e.g. GC-biased gene conversion) as the rest of the genome. The authors show that, compared to the rest of the genome, genes in the UV regions have introns that are shorter, GC-poorer, and have weaker matches to consensus sequences around intron splice sites and branch sites. They also show that nucleosome occupancy and DNA methylation patterns are different between the UV region and the rest of the genome. They propose that these genomic changes are the result of the suppression of recombination in the UV region, and that the genomic changes have in turn led to a big increase in dysfunctional splicing. Nevertheless, the authors argue that the genes in the UV region are still functional: they still make a sufficient level of correctly spliced transcripts. They contrast this "transcriptional degradation" of the UV region with the degradation of genes on (for example) the mammalian Y chromosome, which has occurred mostly by pseudogenization or gene deletion.

The strong aspect of the manuscript is that the results it presents are, as far as I know, completely novel and unexpected, and it is a remarkable discovery. The manuscript has competent data analysis and discussion of the implications. I am aware of previous papers that reported that the UV regions of Mamiellales algae are very GC-poor, but not of ones that reported aberrant splicing in them, so I think this manuscript has high novelty.

The weak aspect of the manuscript is that the text is too brief. It does not provide sufficient background information for readers who are not already very familiar with Mamiellales genomes and their mating type regions. The text, methods and figures/tables also sometimes provide insufficient detail about how some analyses were conducted.

The authors didn't provide line or page numbering. I've numbered the pages starting with the cover page as P1. I use § for paragraph numbers (including for parts of paragraphs, but excluding headers), and L for line number within a paragraph.

==

Major comments:

The Introduction fails to present some basic background information about the UV regions of Mamiellales algae:

-How large are these regions?

-How many genes do they contain?

-Is their gene density, and the number of introns per gene, the same as in the rest of the genome?

-How do we know where the ends of the non-recombining regions are?

-In each species, how similar is the U region to the V region? Are they completely different, or are there U/V paralogous gene pairs?

-Is it thought that all the genes in the UV regions participate in mating functions, or are there also housekeeping genes in these regions?

-Is the manuscript based on analysis of both the U and the V region from each species, or only one of these? Do we know whether U and V both show the same properties (GC-poorness, intron retention, etc)?

The authors examined 4 species of Mamiellales. Are the UV regions of these 4 species orthologous? The word chromosome (singular) in the manuscript's title implies that they are orthologous, as does the text at the end of P3 §3, but the text seem to doesn't cite any paper or data showing that they are orthologs. It is notable from Table S2 that the number of introns in the UV regions differs a lot among the species, so I guess that the number of genes also differs a lot, but the manuscript doesn't report this.

Having read to the end of the Discussion, I am left with some unanswered questions.

-Why is there apparently no selection to relocate genes whose functions are unrelated to mating, from the UV region to autosomal regions where they would function better?

-Or is there evidence that relocation has happened, e.g. some genes in the UV region of one species have autosomal orthologs in a different species?

-Is there evidence that other gene degeneration processes, e.g. pseudogenes, are occurring in the UV regions?

==

Minor comments

P2 §1 (Abstract) L5: The text in many places describes mRNA isoforms as "truncated", but I'm not sure that this is the correct word, as opposed to "aberrant" or simply "intron-retaining". Looking at the example in Figure 3D, most of the transcript isoforms are roughly the same length as the top box, which I guess is the "correct" isoform, and some of them are actually longer because they retain introns.

Figure 1:

-Please provide a definition of "per intron retention frequency" (it's not defined in the Methods section either). Are the plotted points individual introns, or genes?

-It's difficult to see whether, on the chromosomes that contain the UV regions, the frequency of intron retention in the flanking non-UV regions is as low as in the rest of the genome. It would be helpful to comment on this and provide a zoomed-in view of those chromosomes.

Figure 2A and Figure S2: It took me quite a while to realize that the base position called "the fifth position" in the text (P5 §1 L7) is the position labeled "4" in these figures. That's because the numbering in the figures includes a position zero. I suggest re-labeling the X-axis in the figures.

P5 §1: The examples that are mentioned first in the text (i.e. the two exceptions on L3-4, and the G at the fifth position, L7) are quite subtle and difficult to see in Figure 2A/Fig S2. In contrast, the differences at the branch site (L12 and Table S3) are much more dramatic, so why not present them first?

P6 §3 L10: Does this text relate to the Evenness method (P13 §1 L1)? Are the results "data not shown", or is a supplementary table missing?

Figure 3 legend:

-Is this all in M. pusilla?

-In A,B what are the boxes and whiskers? I'm puzzled by the result in panel B for 0-intron genes in non-UV regions - it seems to show a surprisingly large number of truncated CDSs but it has no box.

-In C, what do the thick and thin lines, and the different colors, represent? And the top box? I can guess, but please tell me.

P8 §2 L5 and Table S5:

-Does "80% of the length of the annotated CDS" take any account of the reading frame and translatability? It sounds like you are just considering any transcript that can be aligned at nucleotide level to >80% of the length of the canonical CDS to be functional.

-- Wouldn't it be more pertinent to calculate the fraction of isoforms that can code for a protein >80% of the length of the canonical protein?

-Table S5 has too many decimal places.

-In Table S5, I don't understand the difference between columns 2-4 ("functional ontology terms") and columns 5-7 ("functional domains") and I didn't see any attempt to explain it. Do some domains have ontology terms attached, and others don't?

-The description on P8 §2 L11-15 of an example result from this analysis compares the domain content of *isoforms* from autosomal versus UV regions. Do you really mean isoforms here, rather than transcripts? In other words, would each isoform in Fig 3D be weighted equally, and not by the number of times you detected it in the transcript pool?

P8 §3 L11: Are intergenic distances also systematically shortened?

Table S1: Please add a column showing the ratio between the mean retention level in autosomes versus mating-type regions.

For both Table S2 and Table S3, I'm not as good at mental arithmetic as the authors are, so please add columns showing the frequencies (e.g. in Table S2, in M. pusila, G at the 5th position is at frequency 0.64 in mating-type genes and frequency 0.71 in the non-mating type genes). This would make it easier to see the data supporting the statements in the text at P5 §1.

Table S3: Does "present" indicate that a perfect (6 out of 6) match to the branchpoint sequence is present, or what? Please add a footnote clarifying this. And is the branchpoint sequence shown the consensus sequence from all introns in the genome?

P6 §2 L2 and Figure S3: It's unclear whether the experimental methylation and MNase coverage data comes from a previous publication by another group (ref. 8, cited on P6 §2 L2) or is new data from this study (P11 §5 mentions that chromatin analysis was done for this study).

Figure 4 is not cited anywhere in the text. It looks more like a graphical abstract.

Supplementary Tables and Figures: Please don't put page breaks in the middle of tables, or between a figure and its legend.

P2 §1 (Abstract) L6: By "This suggests…" do you mean "This preservation suggests…"?

P3 §2 L5: Would "genetic decay" not be a better description than "genomic decay"?

P5 §1 L2 citation should be to Figure S2.

P9 §1 should begin "We propose that this collective action…" to make it clear that this paragraph is a continuation of the hypothesis.

P9 §1 L8: Please make it clearer that ref. 12 is a paper about transcription in human genes, not green algal mating type locus genes.

Reviewer #2: The authors perform a comparative transcriptomics analysis across species and genomic region types (mating-type and autosomes), using both publicly available short read RNA sequencing data and long read RNA sequencing that they generate themselves. The first and perhaps most striking result is that intron retention frequencies are considerably higher in mating-type regions than outside (Figure 1), in all 4 species. They analyze frequencies of splicing-regulated motifs and identify several differences between the two categories of genes (including a depletion in canonical branchpoint sequences), suggesting a potential mechanistic basis for the differences observed in intron retention frequencies. Interestingly, intron length and GC content (both of which are lower in mating-type genes) seem to have high predictive power for intron retention frequencies. Both these features are strongly associated with the reduction in recombination rate. Finally, long-read RNA sequencing analysis showed that isoforms in mating type regions are often truncated. All these observations indicate that the increased intron retention rate in mating type regions is due to less efficient splicing, likely through an accumulation of slightly deleterious mutations that cannot be efficiently purged due to reduced recombination.

Overall, I find the manuscript very convincing. I appreciate the fact that the analyses are simple and to the point. The intron retention results are quite striking. I have some suggestions below but overall I am confident that this manuscript is ultimately worthy of publication.

Major comments:

- I'm not sure why the random forest analysis was not performed on autosomal genes. There is definitely lower variation in intron retention rates for this class of genes, but since there are many more genes, a classification could still be interesting. It would be good to know whether the relationships between intron retention on one hand, and GC-content, intron length, expression level etc. are the same for the two categories of genes.

- I have some trouble with the observation that gene expression levels are positively correlated with intron retention rates, across genes in mating type regions. In previous studies performed for a wide variety of species (Saudemont et al, 2018; Bénitière et al, 2024), the opposite correlation between alternative splicing rates and gene expression levels are observed in the great majority of species. Saudemont et al specifically showed that intron retention rates are negatively correlated with gene expression levels in humans and Paramecium. This was explained by the proposal of an increased cost of splicing errors in highly expressed genes. Why would the correlation go the other way around these algal species? I am not an expert in recombination suppresion around mating type loci, so this may be naïve: Could the expression level be anticorrelated with the rate of (historical) recombination in these regions? It would be interesting to perform the same analysis across autosomal genes: can we observe a similar positive correlation between intron retention rates and expression levels?

- Likewise, it would be interesting to test whether the number of introns is associated with intron retention rates in these species, for mating type genes and for autosomal genes. Figure 3 shows the relationship between the number of introns and the proportion of truncated isoforms, but the two observations may be independent.

- What is known about nonsense-mediated decay in these species? I ask this because it seems that properties such as frameshifts and presence of stop codons do not appear to correlate with intron retention rates across mating type genes. It would be good to analyze the rela

- How can one explain the relationship between intron retention rate and position with respect to the gene TSS? If I interpret the graph in Figure 2 correctly (of which I am not sure, cf. comment below), it seems that introns in 3' end of the gene are more often retained. Does this observation hold with long-read sequencing data? Short-read sequencing with polyA selection induces a representation bias towards the end of the transcript, which could induce an estimation bias - retained introns can be more readily detected if the overall coverage is high.

- Finally, do the observations hold for other types of alternative splicing events? One easy way to estimate this is with the alternative variant ratio proposed by Bénitière et al 2024, based on spliced reads. Otherwise one could directly assess exon skipping rates for example.

Minor comments:

- It would be good to show a distribution of gene expression levels within and outside mating type regions in a supplementary figure, to support the analysis on methylation rates.

- In figure 2, B-E, it is not clear how the "partial dependence" on the Y axis should be interpreted. A couple of phrases helping with the interpretation would be helpful.

- This paragraph is not clear:

"Additionally, genes within the mating type region express all isoforms as

a more similar fractions of the total transcript pool than genes in autosomal regions" - do you mean that isoform frequency is less skewed towards one major isoform? This could be clarified.

# Author comment: Author response to Decision Letter 2

**DOI:** 10.1371/journal.pbio.3003823.r003

4 Mar 2026

Attachment Submitted filename: WeirdExpressionMatingTypeManuscript-ResponseDoc.pdf

# Peer review: Decision Letter 2

**DOI:** 10.1371/journal.pbio.3003823.r004

**Authors:** Richard Hodge

8 Apr 2026

Dear Dr Condon,

Thank you for your patience while we considered your revised manuscript "Extensive Splicing Deficiency in a Degenerating Mating-Type Chromosome" for publication as a Short Report at PLOS Biology. Your revised study has been evaluated by the PLOS Biology editors, the Academic Editor and one of the original reviewers (Reviewer #2).

The reviews are attached below. As you can see, Reviewer #2 thinks that the manuscript has been strengthened during revision, but notes that the analysis of additional types of splicing events has not been included as requested. Upon second reading, the reviewer raises potentially important concerns that the observed intron retention could be explained by presence of internal priming due to the presence of AT-rich sequences. He/she suggests conducting long-read sequencing to check whether intron retention is observed at high frequencies when only considering reads that contain a proper polyA tail. After discussions with the academic editor, we agree with these concerns and that additional analysis is required to control for this alternative interpretation.

Given the extent of revision needed, we cannot make a decision about publication until we have seen the revised manuscript and your response to the reviewers' comments. Your revised manuscript is likely to be sent for further evaluation by all or a subset of the reviewers.

In addition to these revisions, you will need to complete some formatting changes, which you will receive in a follow up email. A member of our team will be in touch with a set of requests shortly.

We expect to receive your revised manuscript within 3 months. Please email us (plosbiology@plos.org) if you have any questions or concerns, or would like to request an extension.

At this stage, your manuscript remains formally under active consideration at our journal; please notify us by email if you do not intend to submit a revision so that we may withdraw it.

**IMPORTANT - SUBMITTING YOUR REVISION**

Your revisions should address the specific points made by each reviewer. Please submit the following files along with your revised manuscript:

1. A 'Response to Reviewers' file - this should detail your responses to the editorial requests, present a point-by-point response to all of the reviewers' comments, and indicate the changes made to the manuscript.

*NOTE: In your point-by-point response to the reviewers, please provide the full context of each review. Do not selectively quote paragraphs or sentences to reply to. The entire set of reviewer comments should be present in full and each specific point should be responded to individually, point by point.

You should also cite any additional relevant literature that has been published since the original submission and mention any additional citations in your response.

2. In addition to a clean copy of the manuscript, please also upload a 'track-changes' version of your manuscript that specifies the edits made. This should be uploaded as a "Revised Article with Changes Highlighted" file type.

*Re-submission Checklist*

When you are ready to resubmit your revised manuscript, please refer to this re-submission checklist: https://plos.io/Biology_Checklist

To submit a revised version of your manuscript, please go to https://www.editorialmanager.com/pbiology/ and log in as an Author. Click the link labelled 'Submissions Needing Revision' where you will find your submission record.

Please make sure to read the following important policies and guidelines while preparing your revision:

*Published Peer Review*

Please note while forming your response, if your article is accepted, you may have the opportunity to make the peer review history publicly available. The record will include editor decision letters (with reviews) and your responses to reviewer comments. If eligible, we will contact you to opt in or out. Please see here for more details:

https://blogs.plos.org/plos/2019/05/plos-journals-now-open-for-published-peer-review/

*PLOS Data Policy*

Please note that as a condition of publication PLOS' data policy (http://journals.plos.org/plosbiology/s/data-availability) requires that you make available all data used to draw the conclusions arrived at in your manuscript. If you have not already done so, you must include any data used in your manuscript either in appropriate repositories, within the body of the manuscript, or as supporting information (N.B. this includes any numerical values that were used to generate graphs, histograms etc.). For an example see here: http://www.plosbiology.org/article/info%3Adoi%2F10.1371%2Fjournal.pbio.1001908#s5

*Blot and Gel Data Policy*

We require the original, uncropped and minimally adjusted images supporting all blot and gel results reported in an article's figures or Supporting Information files. We will require these files before a manuscript can be accepted so please prepare them now, if you have not already uploaded them. Please carefully read our guidelines for how to prepare and upload this data: https://journals.plos.org/plosbiology/s/figures#loc-blot-and-gel-reporting-requirements

*Protocols deposition*

To enhance the reproducibility of your results, we recommend that if applicable you deposit your laboratory protocols in protocols.io, where a protocol can be assigned its own identifier (DOI) such that it can be cited independently in the future. Additionally, PLOS ONE offers an option for publishing peer-reviewed Lab Protocol articles, which describe protocols hosted on protocols.io. Read more information on sharing protocols at https://plos.org/protocols?utm_medium=editorial-email&utm_source=authorletters&utm_campaign=protocols

Thank you again for your submission to our journal. We hope that our editorial process has been constructive thus far, and we welcome your feedback at any time. Please don't hesitate to contact us if you have any questions or comments.

Best regards,

Richard

Richard Hodge, PhD

Senior Editor, PLOS Biology

rhodge@plos.org

------------------------------------

REVIEWS:

Reviewer #2: I would like to thank the authors for their answer to my comments. While the manuscript has been improved by the revision, I still have some concerns regarding the manuscript.

1) My suggestion regarding the analysis of other types of alternative splicing events was not addressed. I understand that rMATS cannot properly distinguish intron retention and other AS events, at least in this context. However, one could in fact use other software (or just simple home-made scripts) to analyze alternative splice site usage. Alternative splice sites are detected using exclusively spliced reads. Thus, their detection cannot be affected by intron retention events. This can be easily done with home-made scripts, by analyzing intron coordinates from mapped reads (read mapping software such as HISAT2 can output the coordinates of detected introns).

2) The reason I insist on looking at additional types of alternative splicing events is that, upon rereading the manuscript, I am worried about a potentially important confounding factor: namely, the presence of internal priming due to the presence of AT-rich sequences. The authors note that there are significantly more alternative termination sites in the mating type region. According to their own interpretation, this could be due to mis-priming of oligo-dT primers during library preparation. But this could in fact also explain much of the observed "intron retention". If mis-priming of oligo-dT priming has occurred, it is likely that the sequenced transcriptome contains a non-negligible fraction of transcripts that lack a polyA tail. If a high fraction of the transcripts captured by RNA sequencing in the mating type region are immature, this would lead to an artificial increase in the estimation of intron retention rates. This could also explain the puzzling observation that most retained introns appear towards the 3' end of the transcripts - I don't understand why genuine intron retention would be 3' biased, while inclusion of immature transcripts would indeed generate apparent retained introns in the 3' end of the transcripts. With long read sequencing, one could check whether intron retention is also observed at high frequencies when considering only reads that contain a proper polyA tail. I apologize for not realizing this major issue at the first round of review.

# Author comment: Author response to Decision Letter 3

**DOI:** 10.1371/journal.pbio.3003823.r005

20 Apr 2026

Attachment Submitted filename: Mating Type Manuscript Rev2 Response Doc.docx

# Peer review: Decision Letter 3

**DOI:** 10.1371/journal.pbio.3003823.r006

**Authors:** Richard Hodge

27 Apr 2026

Dear Dr Condon,

Thank you for your patience while we considered your revised manuscript "Extensive Splicing Deficiency in a Degenerating Mating-Type Chromosome" for publication as a Short Report at PLOS Biology. This revised version of your manuscript has been evaluated by the PLOS Biology editors, the Academic Editor and Reviewer #2.

Based on the review from Reviewer #2, I am pleased to say that we are likely to accept this manuscript for publication, provided you satisfactorily address the following data and other policy-related requests that I have provided below:

(A) We routinely suggest changes to titles to ensure maximum accessibility for a broad, non-specialist readership. In this case, we would suggest a minor edit to the title, as follows. Please ensure you change both the manuscript file and the online submission system, as they need to match for final acceptance:

“Splicing deficiency drives genomic erosion in non-recombining algal mating type chromosomes”

(B) You may be aware of the PLOS Data Policy, which requires that all data be made available without restriction: http://journals.plos.org/plosbiology/s/data-availability. For more information, please also see this editorial: http://dx.doi.org/10.1371/journal.pbio.1001797

Note that we do not require all raw data. Rather, we ask that all individual quantitative observations that underlie the data summarized in the figures and results of your paper be made available in one of the following forms:

-Supplementary files (e.g., excel). Please ensure that all data files are uploaded as 'Supporting Information' and are invariably referred to (in the manuscript, figure legends, and the Description field when uploading your files) using the following format verbatim: S1 Data, S2 Data, etc. Multiple panels of a single or even several figures can be included as multiple sheets in one excel file that is saved using exactly the following convention: S1_Data.xlsx (using an underscore).

-Deposition in a publicly available repository. Please also provide the accession code or a reviewer link so that we may view your data before publication.

Regardless of the method selected, please ensure that you provide the individual numerical values that underlie the summary data displayed in the following figure panels as they are essential for readers to assess your analysis and to reproduce it:

Figure 1, 2A-E, 3A-B, S1, S2, S3, S4, S5

NOTE: the numerical data provided should include all replicates AND the way in which the plotted mean and errors were derived (it should not present only the mean/average values).

Please accept my apologies if the underlying data can already be found in your Github deposition. If so, I would be grateful if the labelling of the files could be changed so it is made clearer for the reader which file relates to which specific figure.

(C) Please also ensure that each of the relevant figure legends in your manuscript include information on *WHERE THE UNDERLYING DATA CAN BE FOUND*, and ensure your supplemental data file/s has a legend.

(D) Please note that we cannot accept sole deposition of code in GitHub, as this could be changed after publication. However, you can archive this version of your publicly available GitHub code to Zenodo. Once you do this, it will generate a DOI number, which you will need to provide in the Data Accessibility Statement (you are welcome to also provide the GitHub access information). See the process for doing this here: https://docs.github.com/en/repositories/archiving-a-github-repository/referencing-and-citing-content

------------------------------------------------------------------------

As you address these items, please take this last chance to review your reference list to ensure that it is complete and correct. If you have cited papers that have been retracted, please include the rationale for doing so in the manuscript text, or remove these references and replace them with relevant current references. Any changes to the reference list should be mentioned in the cover letter that accompanies your revised manuscript.

In addition to these revisions, you may need to complete some formatting changes, which you will receive in a follow up email. A member of our team will be in touch with a set of requests shortly. If you do not receive a separate email within a few days, please assume that checks have been completed, and no additional changes are required.

We expect to receive your revised manuscript within two weeks.

To submit your revision, please go to https://www.editorialmanager.com/pbiology/ and log in as an Author. Click the link labelled 'Submissions Needing Revision' to find your submission record. Your revised submission must include the following:

- a cover letter that should detail your responses to any editorial requests, if applicable, and whether changes have been made to the reference list

- a Response to Reviewers file that provides a detailed response to the reviewers' comments (if applicable, if not applicable please do not delete your existing 'Response to Reviewers' file.)

- a track-changes file indicating any changes that you have made to the manuscript.

NOTE: If Supporting Information files are included with your article, note that these are not copyedited and will be published as they are submitted. Please ensure that these files are legible and of high quality (at least 300 dpi) in an easily accessible file format. For this reason, please be aware that any references listed in an SI file will not be indexed. For more information, see our Supporting Information guidelines:

https://journals.plos.org/plosbiology/s/supporting-information

*Published Peer Review History*

Please note that you may have the opportunity to make the peer review history publicly available. The record will include editor decision letters (with reviews) and your responses to reviewer comments. If eligible, we will contact you to opt in or out. Please see here for more details:

https://plos.org/published-peer-review-history/

*Press*

Should you, your institution's press office or the journal office choose to press release your paper, please ensure you have opted out of Early Article Posting on the submission form. We ask that you notify us as soon as possible if you or your institution is planning to press release the article.

*Protocols deposition*

To enhance the reproducibility of your results, we recommend that if applicable you deposit your laboratory protocols in protocols.io, where a protocol can be assigned its own identifier (DOI) such that it can be cited independently in the future. Additionally, PLOS ONE offers an option for publishing peer-reviewed Lab Protocol articles, which describe protocols hosted on protocols.io. Read more information on sharing protocols at https://plos.org/protocols?utm_medium=editorial-email&utm_source=authorletters&utm_campaign=protocols

Please do not hesitate to contact me should you have any questions.

Best regards,

Richard

Richard Hodge, PhD

Senior Editor, PLOS Biology

rhodge@plos.org

------------------------------------------------------------------------

Reviewer remarks:

Reviewer #2: I would like to thank the authors for their effort addressing my last comment. I have no further remarks.

# Author comment: Author response to Decision Letter 4

**DOI:** 10.1371/journal.pbio.3003823.r007

30 Apr 2026

Attachment Submitted filename: Mating_Type_Manuscript_Rev2_Response_Doc_auresp_4.docx

# Editor report: Decision Letter 4

**DOI:** 10.1371/journal.pbio.3003823.r008

**Authors:** Richard Hodge

12 May 2026

Dear Chris,

On behalf of my colleagues and the Academic Editor, Laurence Hurst, I am pleased to say that we can accept your manuscript for publication, provided you address any remaining formatting and reporting issues. These will be detailed in an email you should receive within 2-3 business days from our colleagues in the journal operations team; no action is required from you until then. Please note that we will not be able to formally accept your manuscript and schedule it for publication until you have completed any requested changes.

Please take a minute to log into Editorial Manager at http://www.editorialmanager.com/pbiology/, click the "Update My Information" link at the top of the page, and update your user information to ensure an efficient production process.

PRESS

We frequently collaborate with press offices. If your institution or institutions have a press office, please notify them about your upcoming paper at this point, to enable them to help maximise its impact. If the press office is planning to promote your findings, we would be grateful if they could coordinate with biologypress@plos.org. If you have previously opted in to the early version process, we ask that you notify us immediately of any press plans so that we may opt out on your behalf.

We also ask that you take this opportunity to read our Embargo Policy regarding the discussion, promotion and media coverage of work that is yet to be published by PLOS. As your manuscript is not yet published, it is bound by the conditions of our Embargo Policy. Please be aware that this policy is in place both to ensure that any press coverage of your article is fully substantiated and to provide a direct link between such coverage and the published work. For full details of our Embargo Policy, please visit http://www.plos.org/about/media-inquiries/embargo-policy/.

Thank you again for choosing PLOS Biology for publication and supporting Open Access publishing. We look forward to publishing your study.

Best wishes,

Richard

Richard Hodge, PhD

Senior Editor, PLOS Biology

rhodge@plos.org

PLOS

Empowering researchers to transform science

Carlyle House, Carlyle Road, Cambridge, CB4 3DN, United Kingdom

California (U.S.) corporation #C2354500, based in San Francisco
